# 

**Published:** 1887-11

**Authors:** 


### CONTENTS, NOV. 1887, (VOL. III, NO. V.)

ORIGINAL COMMUNICATIONS.—Original Observations and Methods in the Practice of Medicine: by L. M. Stroud, M. D., Forney, Texas...................... 163

Congestion of the Brain, followed by Amnesic Aphasia: by A. H. McCord, M. D., Rusk, Texas............... 171

Ear-Pain, and its Treatment: by E. Mierhof, M. D., New York.................................... 172

BIOGRAPHICAL.—Thomas Dudley Wooten, M. D., President Austin Dist. Med. Soc., etc..................... 175

SOCIETY NOTES.—The Southern Surgical and Gynecological Association................................ 179

Austin District Medical Society....................... 180

To Regulate the Practice in all the States.............. 181

CORRESPONDENCE.—A new remedy for Asthma; letter from Dr. L. J. Graham, Henderson, Texas........... 182

Legal Test of Claims for Medical Services at Inquest, and Expert Testimony: Reported by Dr. J. E. Mayfield, Nacogdoches, Texas......................... 183

FOREIGN CORRESPONDENCE.—Letter from Our own Correspondent in Paris, Dr. Weymann.................... 185

EDITORIAL—The Vexed Question of Gleditschine........ 188

A Scientific Achievement of Practical Value.......... 192

Dr. Paine's Discoveries.............................. 194

The Texas Special Committee in Surgery............. 195

"Squibbian Inconsistency"......................... 196

MEDICAL NEWS AND MISCELLANY.—Antipyrine; The Crown Prince of Germany. Death of Dr. Johnson; Our Paris Letter; the Crowning Achievement of Ophthalmic Surgery; the Stenocapine Sensation; Austin Sanitarium; Acquitted; Lehn & Fink; The Nacogdoches Test case; A Cure for Wrinkles; Death of Prof. Gunn; A Doctor in Limbo; Gaillard's Medical Journal; Biography Contemporary Physicians of Texas; Errata, Colotomy in Rectal Disease.............................. 197-205

ADVERTISERS' NOTICES................................ 205

